# ADAMTS10-mediated tissue disruption in Weill–Marchesani syndrome

**DOI:** 10.1093/hmg/ddy276

**Published:** 2018-07-27

**Authors:** Ewa J Mularczyk, Mukti Singh, Alan R F Godwin, Francessco Galli, Neil Humphreys, Antony D Adamson, Aleksandr Mironov, Stuart A Cain, Gerhard Sengle, Ray P Boot-Handford, Giulio Cossu, Cay M Kielty, Clair Baldock

**Affiliations:** 1 Wellcome Centre for Cell Matrix Research, Division of Cell-Matrix Biology and Regenerative Medicine, School of Biological Sciences, Faculty of Biology, Medicine and Health, University of Manchester, Manchester Academic Health Science Centre, UK; 2 Division of Cell-Matrix Biology and Regenerative Medicine, School of Biological Sciences, Faculty of Biology, Medicine and Health, University of Manchester, UK; 3 School of Biological Sciences, Faculty of Biology, Medicine and Health, University of Manchester, UK; 4 Center for Biochemistry, Center for Molecular Medicine (CMMC), Medical Faculty, University of Cologne, Germany

## Abstract

Fibrillin microfibrils are extracellular matrix assemblies that form the template for elastic fibres, endow blood vessels, skin and other elastic tissues with extensible properties. They also regulate the bioavailability of potent growth factors of the TGF-β superfamily. A disintegrin and metalloproteinase with thrombospondin motifs (ADAMTS)10 is an essential factor in fibrillin microfibril function. Mutations in fibrillin-1 or ADAMTS10 cause Weill–Marchesani syndrome (WMS) characterized by short stature, eye defects, hypermuscularity and thickened skin. Despite its importance, there is poor understanding of the role of ADAMTS10 and its function in fibrillin microfibril assembly. We have generated an ADAMTS10 WMS mouse model using Clustered Regularly Spaced Interspaced Short Palindromic Repeats and CRISPR associated protein 9 (CRISPR-Cas9) to introduce a truncation mutation seen in WMS patients. Homozygous WMS mice are smaller and have shorter long bones with perturbation to the zones of the developing growth plate and changes in cell proliferation. Furthermore, there are abnormalities in the ciliary apparatus of the eye with decreased ciliary processes and abundant fibrillin-2 microfibrils suggesting perturbation of a developmental expression switch. WMS mice have increased skeletal muscle mass and more myofibres, which is likely a consequence of an altered skeletal myogenesis. These results correlated with expression data showing down regulation of Growth differentiation factor (GDF8) and Bone Morphogenetic Protein (BMP) growth factor genes. In addition, the mitochondria in skeletal muscle are larger with irregular shape coupled with increased phospho-p38 mitogen-activated protein kinase (MAPK) suggesting muscle remodelling. Our data indicate that decreased SMAD1/5/8 and increased p38/MAPK signalling are associated with ADAMTS10-induced WMS. This model will allow further studies of the disease mechanism to facilitate the development of therapeutic interventions.

## Introduction

A disintegrin and metalloproteinase with thrombospondin motifs (ADAMTS) proteins are members of a large superfamily of 19 secreted ADAMTS proteases and 7 ADAMTS-like proteins (1). The multi-domain zinc metalloproteases each have an N-terminal catalytic and disintegrin-like domain and C-terminal region that contains thrombospondin repeats that may interact with the extracellular matrix. Secreted as zymogens, the ADAMTS proteases are activated at the cell surface by cleavage of the N-propeptide by furin or other convertases. However, ADAMTS10 is resistant to furin cleavage due to lack of conservation of the furin recognition sequence and does not appear to be catalytically active (2). The ADAMTS/L family is involved in many biological functions including angiogenesis, coagulation, morphogenesis and development, as well as diseases such as cancer, arthritis and extracellular matrix disorders (3). Mutations in ADAMTS10 (autosomal recessive) (4,5) have been linked to Weill–Marchesani Syndrome (WMS), a rare genetic disorder with an estimated prevalence of 1:100 000 people. Clinical features include short stature, microspherophakia, ectopia lentis (lens dislocation), hypermuscularity, thickened skin, brachydactyly and stiffened joints (4,6).

Mutations in fibrillin-1 (Fbn1) (autosomal dominant) also cause WMS (7–9) which implied a role for ADAMTS10 in Fbn1 assembly and indicates dual roles for their functions in the pathways disrupted in WMS (10). Fibrillin microfibrils are evolutionarily ancient extracellular matrix assemblies that form the template for elastic fibres that endow blood vessels, lungs, skin, ligaments and other elastic tissues with essential extensible properties. They also regulate the bioavailability of potent growth factors of the TGFβ and BMP superfamily (11,12). Mutations in Fbn1 most commonly cause Marfan syndrome (MFS) with life-threatening cardiovascular defects, bone overgrowth, joint laxity/contractures and ectopia lentis, caused by elastic fibre defects and/or growth factor dysregulation. However, a subset of Fbn1 mutations in two specific regions of the Fbn1 molecule have also been identified that cause WMS (8), which has many opposing clinical features to MFS but ectopia lentis in common with MFS (5).

Subsequently, ADAMTS10 has been shown to be a regulator of fibrillin microfibril function, ADAMTS10 binds Fbn1 at two sites which coincide with the Fbn1 WMS mutations (2), co-localizes with Fbn1 and accelerates microfibril assembly in fibroblast cultures (8). In agreement, we found that ADAMTS10 depletion reduced microfibril deposition. We have shown that ADAMTS10 supports epithelial cell–cell junctions, binds heparan sulphate (HS) and supports focal adhesion formation (13). ADAMTS6 (an active homologue of ADAMTS10) (14) strongly binds HS and can cleave Fbn1 and syndecan-4 (13). We also found that the Fbn1 TB5 domain (which harbours WMS mutation sites) binds HS (15), and that all disease-causing mutations tested disrupt this interaction (16), implicating HS in these fibrillinopathies. We have also shown that ADAMTS10 is needed for ZO-1-rich tight junction integrity in epithelial cells; in contrast, ADAMTS6 depletion enhances tight junctions and ADAMTS10 negatively regulates ADAMTS6 expression (13).

Other ADAMTS and ADAMTS-like proteins have been linked to fibrillin biology, either genetically by association with fibrillinopathies or functionally (17). ADAMTS17 and 19 are also co-localized with microfibrils in the extracellular matrix. Mutations in ADAMTS17 cause WMS-like syndrome with ectopia lentis (17) and ADAMTSL4 mutations cause isolated ectopia lentis and ectopia lentis et pupillae (18). ADAMTSL6 can also bind Fbn1, and it enhances microfibril formation *in vitro* and in over-expressing transgenic mice (19), indicating roles for a number of ADAMTS and ADAMTSL molecules in fibrillin microfibril assembly and function, although the mechanisms are as yet unknown.

Here we have generated an ADAMTS10 mouse with mutation similar to the R237X WMS-causing mutation observed in patients. The mice recapitulate the short-stature phenotype of patients and show developmental changes to the growth plate. Moreover, abnormalities to the ciliary apparatus are observed coupled with altered distribution of Fbn1 and fibrillin-2 (Fbn2) microfibrils. The mutant mice have increased skeletal muscle mass and muscle cells show signs of muscle regeneration/stress with decreased BMP and increased MAPK signalling.

## Results

**Figure 1:**
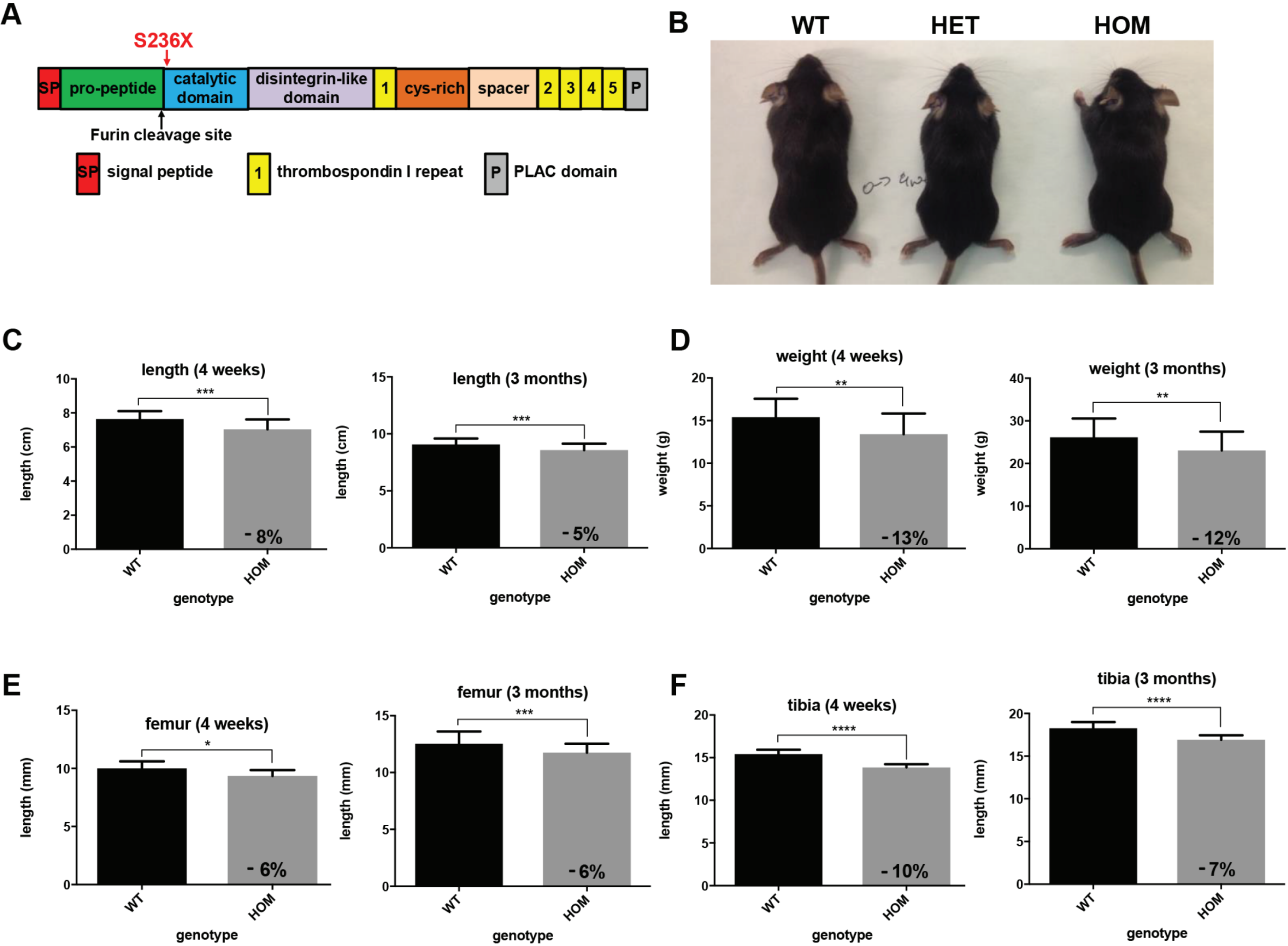
The ADAMTS10^S236X/S236X^ mouse recapitulates WMS short-stature phenotype. **(A)** Domain map of ADAMTS10. Red arrow shows localization of S236X mutation. Black arrow shows location of partially conserved furin cleavage site (^230^GLKR^233^). **(B)** 4 week-old wild-type and WMS ADAMTS10^S236X/S236X^ mice. Body measurements for the WT and HOM 4-week-old and 3 month-old mice with graphs representing **(C)** length and **(D)** weight, n = 16. Graphs representing **(E)** length of 4 week-old and 3 month-old WT and HOM mouse tibia and **(F)** femur, n ≥ 6. Statistical significance was calculated using two-tailed unpaired Student’s test in GraphPad Prism V6. Asterisk indicate *P***-**values where ^*^P-value ≤ 0.05; ^**^P-value ≤ 0.01;^ ***^P-value ≤ 0.001; ^****^P-value ≤ 0.0001.

**Figure 2:**
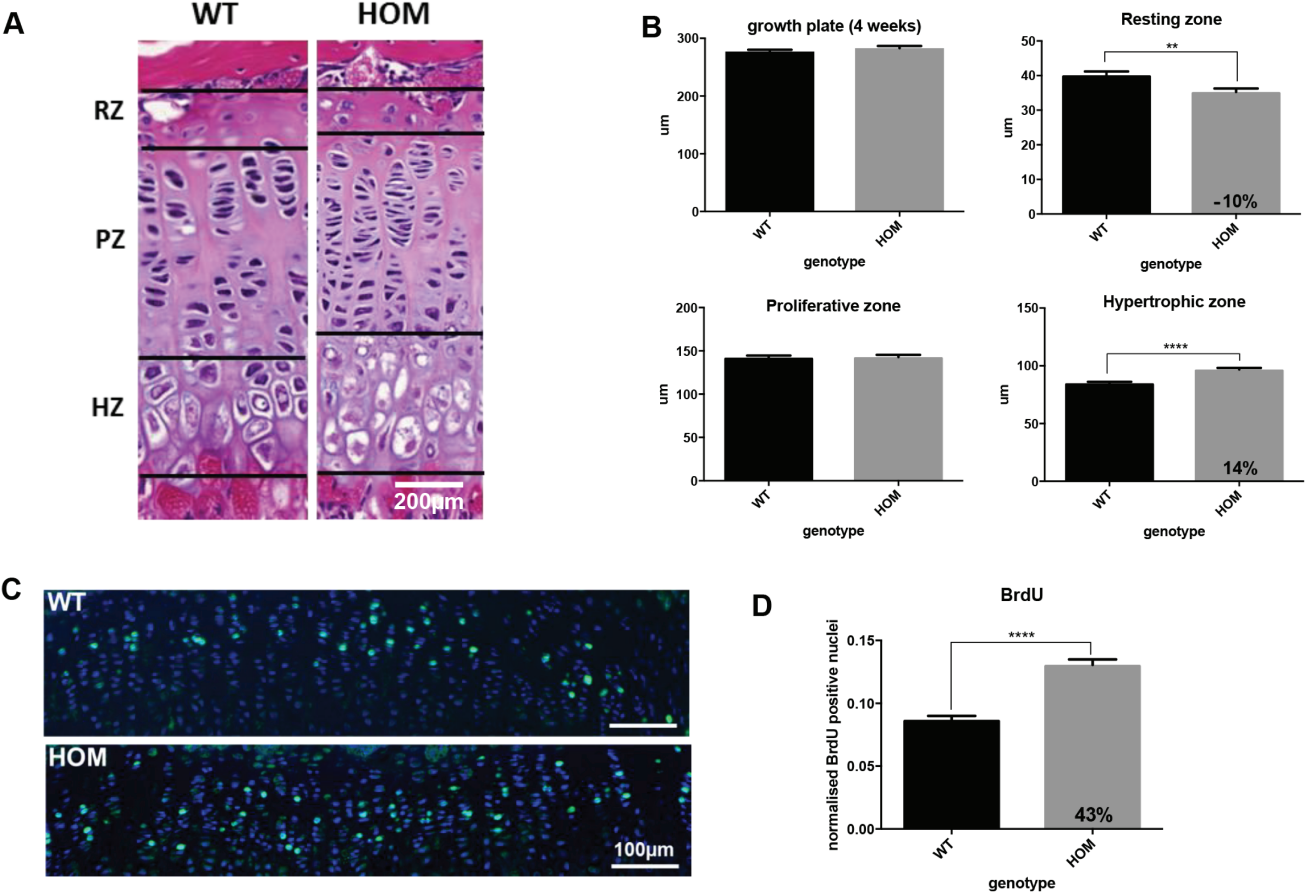
Altered proliferation and bone development in WMS ADAMTS10^S236X/S236X^ mice. **(A)** H&E staining of WT and HOM growth plates and the zones: RZ, proliferating zone (PZ) and HZ showing altered bone development of the mutant mice at 3 weeks; n = 3, scale bar = 200 μm. **(B)** Quantification of H&E stained images as shown in (A), n = 3, for each mouse at least 3 measurements of the growth plate and zones were taken from 10 consecutive 5 μm-spaced sections (>30 per mouse). (**C)** DAPI (blue) and 5-bromo-29-deoxyuridine (BrdU, green) labelling of proliferative cells in the growth plate of 3-week-old WT and HOM mice. Positive cells stained green. Scale bar = 100 μm. **(D)** Graph showing an increased percentage of BrdU positive cells within the proliferative zone in the mutant sample. The number of labelled cells in 10 different sections from 3 WT and 3 HOM animals were counted. Statistical significance was calculated using two-tailed unpaired Student’s test in GraphPad Prism V6. Asterisk indicate *P*-values where ^*^P-value ≤ 0.05; ^**^P-value ≤ 0.0; ^****^P-value ≤ 0.0001.

We have generated a WMS mouse model with S236X mutation in ADAMTS10 which is similar to the R237X mutation identified in WMS patients (4) using CRISPR-Cas9. The mutation site (S236) is immediately after the propeptide at the start of the catalytic domain (Arg233) (Fig. 1A; Supplementary Fig. 1). The ADAMTS10^S236X/S236X^ homozygous mice are smaller than wild-type littermates, which is apparent from both their weight and length measurements and shown at 4 weeks and 3 months (Fig. 1B–D). The long bones of the ADAMTS10^S236X/S236X^ homozygous mice are 6–10% shorter than wild-type mice as shown for femur and tibia (Fig. 1E–F). Staining of the growth plate shows a normal structure but with differences in the relative proportions of the zones with shorter resting zone (RZ) and expanded hypertrophic zone (HZ) suggesting altered bone development in the mutant mice (Fig. 2). ADAMTS10 is expressed in the knee joint, predominantly in the articular cartilage, perichondrium and meniscus but also in the growth plate and secondary ossification centre (Supplementary Fig. 2). To analyse any changes in cell proliferation within the growth plate of 3-week-old mice, bromodeoxyuridine (BrdU) labelling was performed showing an increased percentage of BrdU positive cells within the proliferative zone in the mutant suggesting increased proliferation (Fig. 2C  and D). Taken together, these data suggest that chondrocyte differentiation in the growth plate is altered.

**Figure 3:**
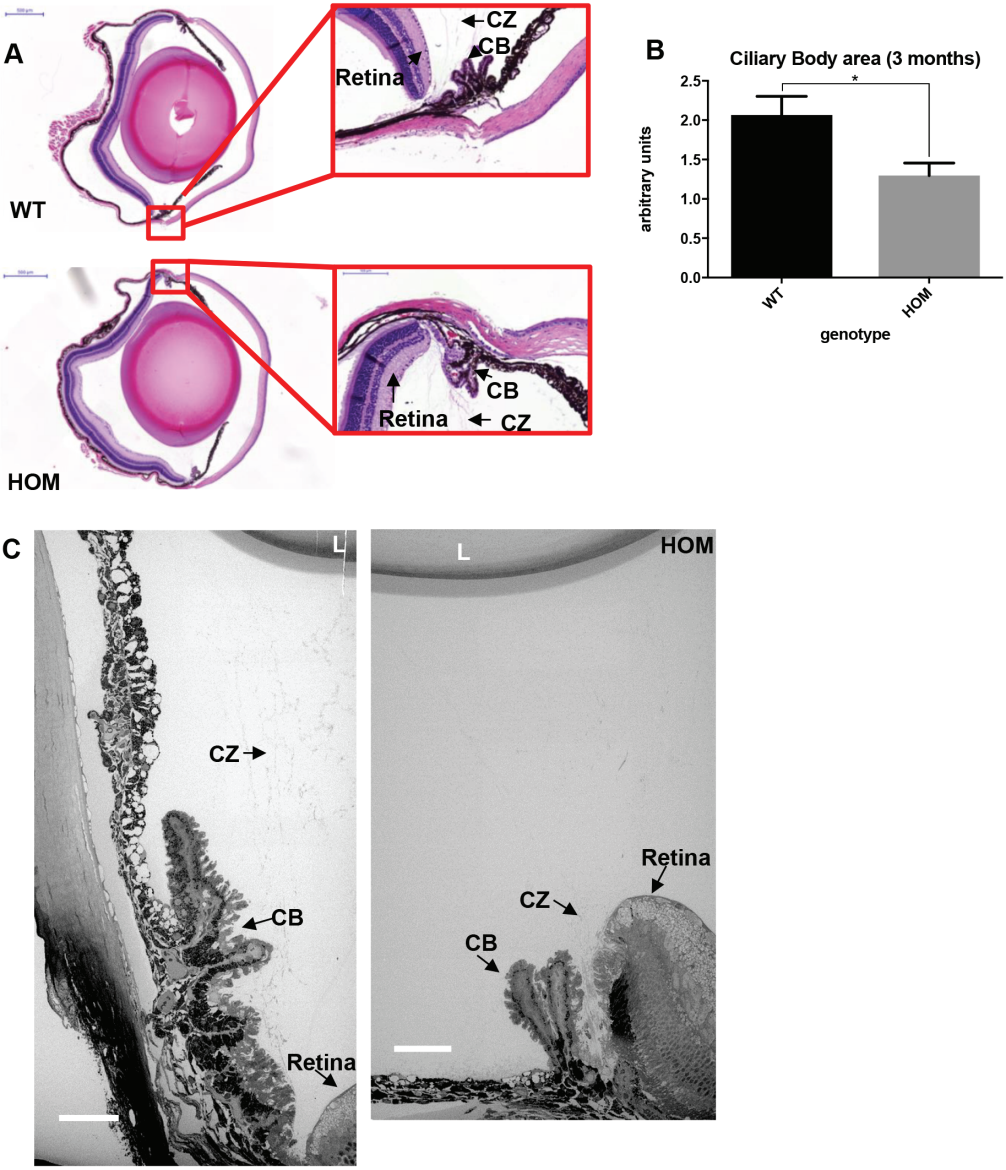
Abnormal ciliary processes in the ADAMTS10^S236X/S236X^ mouse. **(A)** H&E stained sections from 3-month-old WT and HOM mouse eyes, insets with ciliary bodies, scale bars = 500 μm (left panel) and 100 μm (right panel). The retina, CZ and CB are labelled. **(B)** Quantification of CB area in the WT and HOM samples showing decreased CB area in the latter, n = 6. **(C)** WT and HOM CZ was imaged using SBF-SEM, scale bar = 50 μm, lens (L). Statistical significance was calculated using two-tail unpaired Student’s test in GraphPad Prism V6. Asterisk indicate *P*-values where ^*^P-value ≤ 0.05.

**Figure 4:**
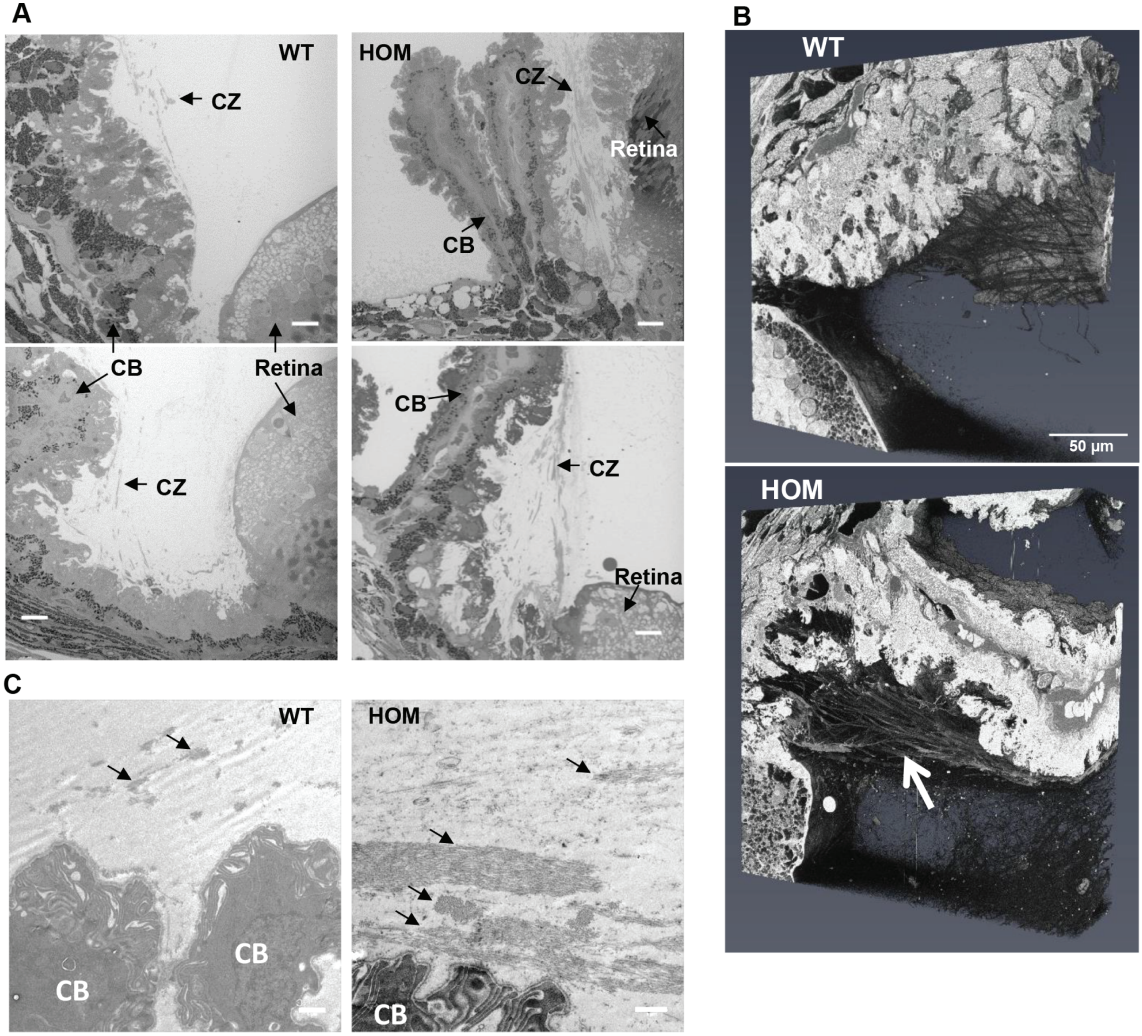
Increased microfibril bundles in CZs adjacent to the CB. **(A)** 3-month-old WT and HOM CZs were imaged using serial block face scanning electron microscopy, n = 3. The retina, CZ and CB are labelled. Scale bar = 10 μm. **(B)** 3D volumes of the regions shown in (A) with the white arrow indicating large bundles of microfibrils emerging from the ciliary epithelium. **(C)** TEM images showing microfibril bundles indicated by black arrows, many larger bundles are present in the HOM eye compared to the wild type, scale bar = 1 μm.

Since WMS patients present with ectopia lentis and other eye disorders, the distribution of ADAMTS10 in the eye was analysed. Since ADAMTS10 protein localization in the eye has not been shown previously, immunofluorescent staining was performed showing broad distribution of ADAMTS10 in the structures of the adult eye, including the ciliary body (CB), cornea, ciliary zonule (CZ) and non-pigmented epithelium (Supplementary Fig. 3A). Therefore, the anatomy of the ADAMTS10^S236X/S236X^ eyes was examined. Haematoxylin and Eosin (H&E) staining showed disruption to the CB with changes in the number and shape of the ciliary processes and a reduction in the area of the ciliary apparatus (Fig. 3A and B). The reduction in the area of the ciliary apparatus was confirmed using serial blockface SEM imaging with low magnification images (Fig. 3C) demonstrating that the CB in the mutant mice is smaller and has fewer ciliary processes. This finding is consistent with observations that WMS patients have smaller ciliary bodies (20). The CB also seems to be joined to, or is closer to, the retina (Fig. 3C). Higher magnification images show that the CZ of the homozygous mutant mice also appears to be altered when compared to the CZ of the WT mice (Fig. 4A). In the CZ adjacent to the CB, the ADAMTS10^S236X/S236X^ mice have many more microfibril bundles than the wild-type mouse (Fig. 4). Larger and thicker bundles can be seen to emerge from the CB and also clearly apparent in the 3D reconstructions and Transmission electron microscope (TEM) images shown in Figure 4B and C. However, the morphology of individual microfibrils purified from the skin of ADAMTS10^S236X/S236X^ mice is normal so ADAMTS10 is not required for assembling individual microfibrils (Supplementary Fig. 4).

**Figure 5:**
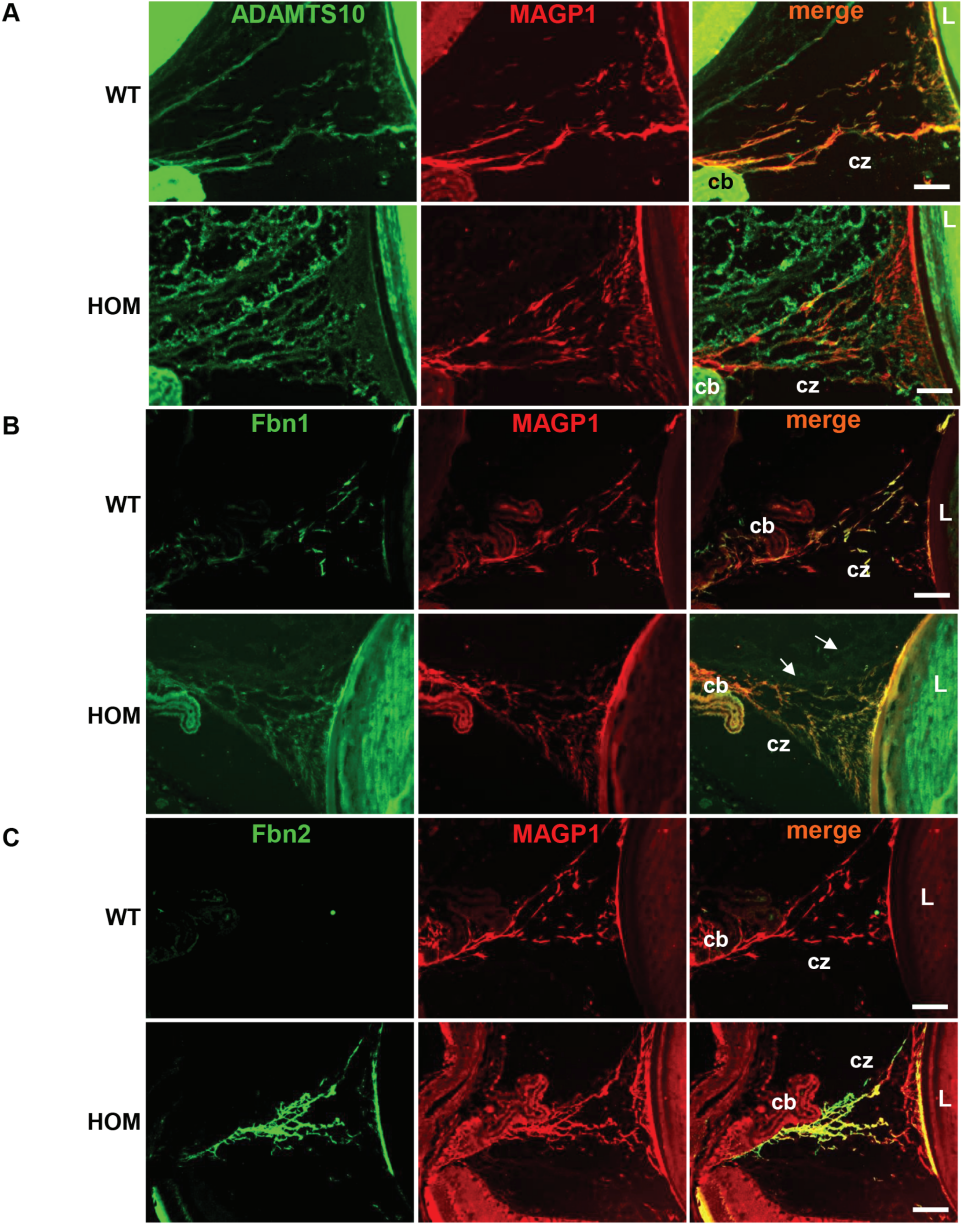
Secretion of mutant ADAMTS10 and increased expression of Fbn2 in the CZs. Immunohistochemical staining in the 3-month-old WT and HOM CZ of **(A)** ADAMTS10 (green) and MAGP1 (red) showing reduced co-localization in the mutant samples. **(B)** FBN1 (green) and MAGP1 (red) fully co-localize in the WT CZ, however in the mutant samples there are FBN1 disorganized deposits observed outside of the CZ (white arrows). **(C)** FBN2 (green) is absent in the WT sample, however it is detected and co-localizes with MAGP1 (red) in the mutant CZ. Scale bars = 50 μm, CB, CZ, L. Negative control images are shown in Supplementary Figure 3.

In order to determine the composition of the CZ fibres, immunohistochemical staining in the 3-month-old wild-type and ADAMTS10^S236X/S236X^ CZ showed co-localization of ADAMTS10 and MAGP1 (a fibrillin microfibril associated protein and marker for microfibrils) in the wild type. However, in the ADAMTS10^S236X/S236X^ CZs there is reduced co-localization but more mutant protein accumulated in disorganized foci (Fig. 5A). Fbn1 and MAGP1 co-localize in the wildtype (WT) CZ and the mutant CZs appear largely unchanged in terms of the Fbn1 staining, however there are some deposits of Fbn1 observed outside of the CZ (Fig. 5B). Fbn2, which is mainly expressed during development, is absent in the WT sample. However abundant Fbn2 microfibrils are present in the mutant CZ and co-localize with MAGP1 (Fig. 5C) suggesting that the increased microfibril bundles observed in Figure 4 are accumulations of Fbn2 microfibrils rather than Fbn1.

**Figure 6:**
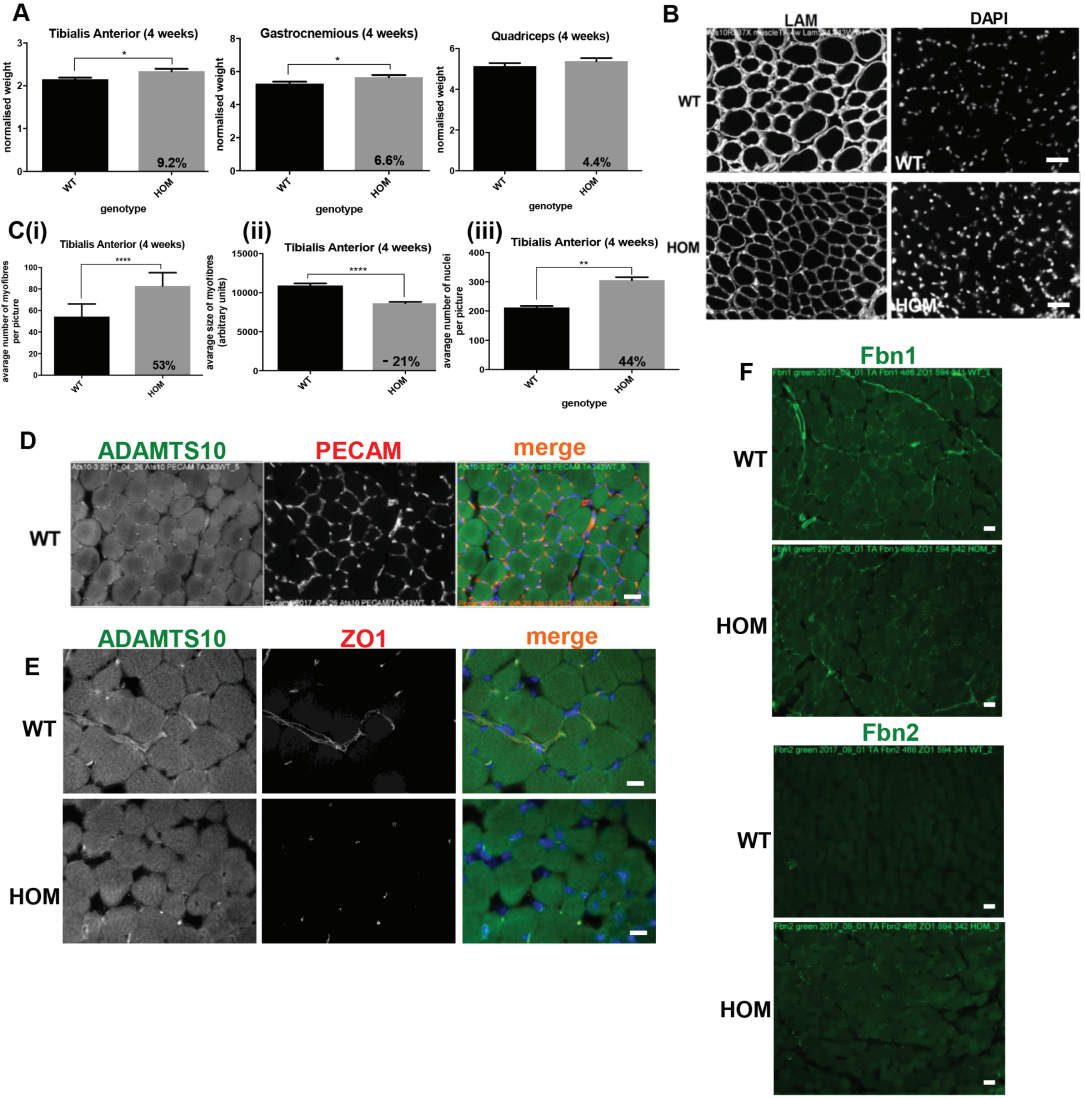
ADAMTS10 in muscle niche and disruption of ZO-1 tight junctions. **(A)** Graphs representing normalized weight of the 4-week-old WT and HOM TA, Gastrocnemius (G) and Quadriceps (Q). **(B)** IF staining of laminin (LAM) and nuclei in the WT and HOM TA. **(C)** Quantification of (i) number and (ii) size of myofibres and (iii) number of nuclei in the 4-week TA, n ≥ 6. **(D)** IF staining of PECAM1 (red) and ADAMTS10 (green) in wild-type TA. **(E)** IF staining of ADAMTS10 (green) and tight junctions (ZO-1, red) in TA showing loss of the latter in HOM muscle. **(F)** IF staining of Fbn1 and Fbn2 (green) in TA showing presence of Fbn1 in both WT and HOM and Fbn2 in HOM muscle only, scale bars = 50 μm. Negative control images are shown in Supplementary Figure 5. Statistical significance was calculated using two-tail unpaired Student’s test in GraphPad Prism V6. Asterisk indicate *P*-values where ^*^P-value ≤ 0.05; ^**^P-value ≤ 0.01; ^****^P-value ≤ 0.0001.

WMS patients are reported to be hypermuscular and indeed the ADAMTS10^S236X/S236X^ mice have increased skeletal muscle mass when normalized to body weight as shown for 4-week-old mice (Fig. 6A). The skeletal muscle of the ADAMTS10^S236X/S236X^ mice has increased number of myofibres and increased nuclei, but the myofibres are smaller (Fig. 6B and C). Staining with the endothelial marker Platelet endothelial cell adhesion molecule (PECAM) and Cluster of differentiation 31 (CD31) (PECAM/CD31) shows that ADAMTS10 partially co-localizes with the muscle niche in the wild-type mouse (Fig. 6D). Previously, we showed that ADAMTS10 is needed for ZO-1-rich tight junction integrity in epithelial cells. Indeed in the Tibialis anterior (TA) ADAMTS10 localizes to ZO-1-rich tight junctions, which are reduced in the ADAMTS10^S236X/S236X^ muscle (Fig. 6E). In addition, whereas Fbn1 is detected in both WT and Homozygous (HOM) muscle, Fbn2 is only present in the mutant tissue (Fig. 6F). This suggests that, similar to the CZs, the switch between Fbn2 and Fbn1 is altered and is a general phenomenon rather than a tissue specific mechanism.

**Figure 7:**
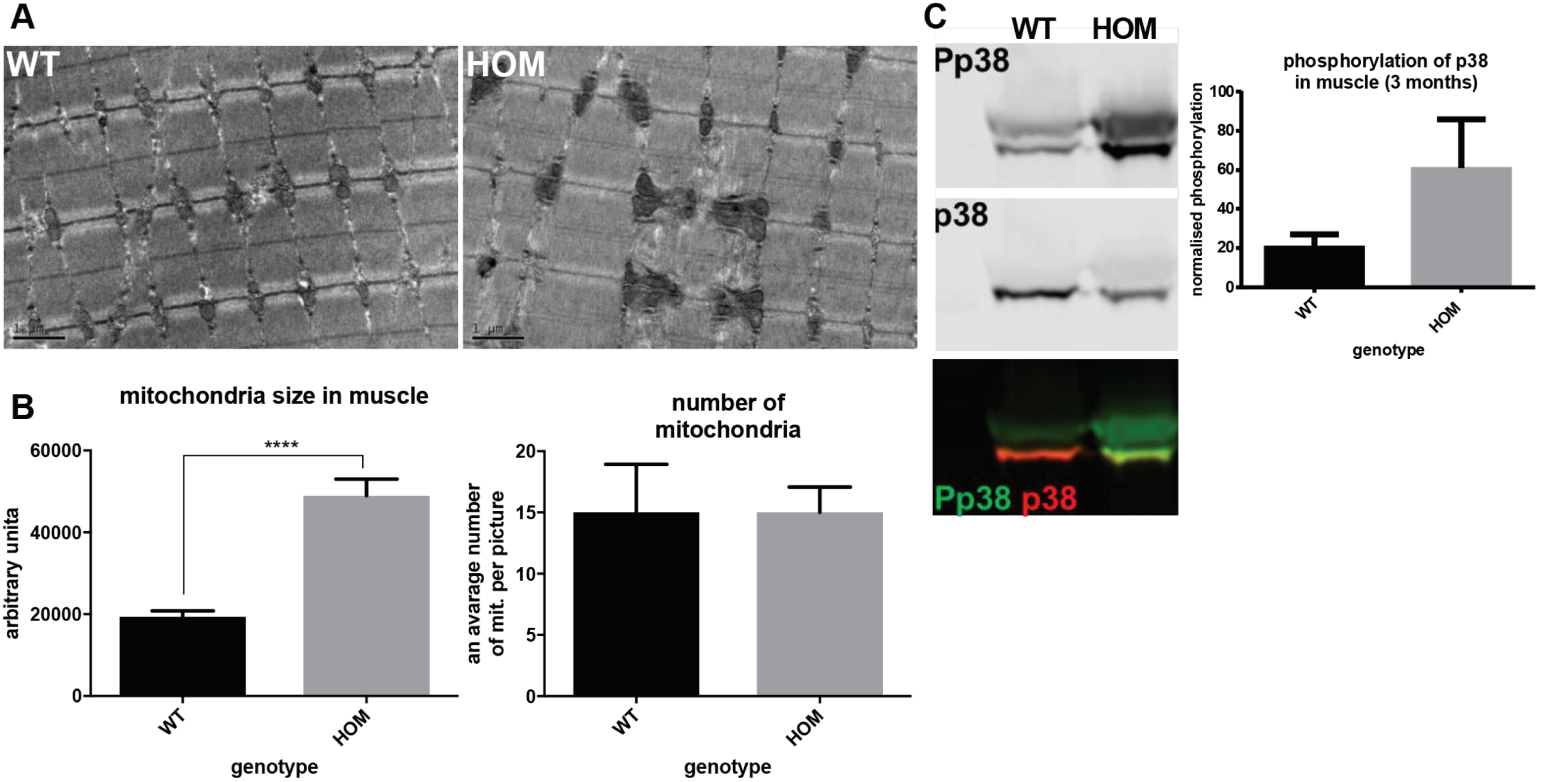
Perturbed skeletal muscle homeostasis in WMS ADAMTS10^S236X/S236X^muscle. **(A)** TEM of the 3-month-old WT and HOM muscle tissue showing enlarged mitochondria in the latter, scale bar = 1 μm. **(B)** Quantification of size and number of mitochondria (one mouse 4 areas). **(C)** Western blots of phospho-p38 (Pp38) and p38 protein levels in the 4-week-old skeletal muscle lysates showing increased MAPK signalling in the WMS muscle. Normalized quantifications, n = 3. Statistical significance was calculated using two-tail unpaired Student’s test in GraphPad Prism V6. Asterisk indicate *P*-values where ^****^P-value ≤ 0.0001.

**Figure 8:**
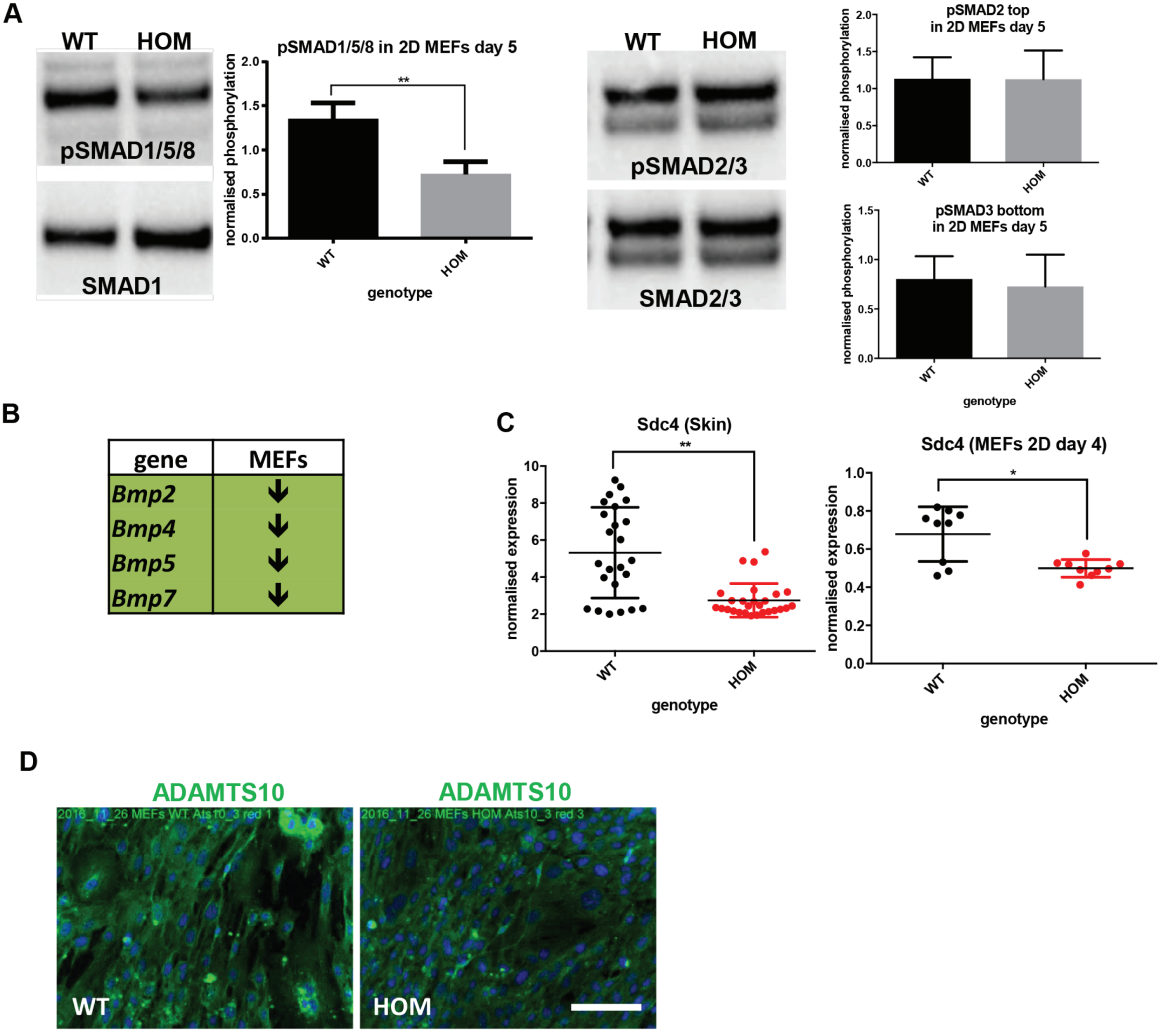
Reduced BMP signalling and syndecan-4 expression in ADAMTS10^S236X/S236X^ skin and MEFs. **(A)** pSMAD1/5/8, SMAD1 and pSMAD2/3, SMAD2/3 (the top band is (p)SMAD2 and the lower band (p)SMAD3) in MEFs isolated from the WT and HOM embryos cultured in 2D for 5 days. Normalized quantifications, n = 3. **(B)** RT-PCRs shows down-regulation of Bmp signalling in HOM 4 day 2D MEFs (experimental data shown in Supplementary Fig. 6). **(C)** RT-PCR data showing decreased syndecan-4 expression in both HOM 3-month-old mouse skin and 4 day 2D MEFs, n = 3. **(D)** IF microscopy of the WT and HOM MEFs cultured for 4 days and stained with ADAMTS10 (green) and DAPI (blue), negative staining controls are included in Supplementary Figure 8. Scale bar = 100 μm. Statistical significance was calculated using two-tail unpaired Student’s test in GraphPad Prism V6. Asterisk indicate *P*-values where ^*^P-value ≤ 0.05; ^**^P-value ≤ 0.01; ^***^P-value ≤ 0.001; ^****^P-value ≤ 0.0001.

Expression data show a number of changes in the ADAMTS10^S236X/S236X^ muscle including up regulation of ADAMTS6 (Supplementary Fig. 6), supporting our published finding that ADAMTS10 negatively regulates ADAMTS6 expression (13). Since ADAMTS6 depletion enhances tight junctions this could further contribute to the reduction of ZO-1 tight junctions in the mutant. GDF8/myostatin is also down regulated in the ADAMTS10 mutant, a negative regulator of muscle growth. Quantitative Polymerase Chain Reaction (qPCR) gene expression data also show that the ADAMTS10 transcript is present in ADAMTS10^S236X/S236X^ mice (Supplementary Fig. 6). This indicates that the Messenger ribonucleic acid (mRNA) does not undergo nonsense-mediated decay and can be translated into the truncated protein, which is confirmed in Figs. 5A ,6E and 8D. Furthermore, the muscle cells of the ADAMTS10^S236X/S236X^ mice have enlarged abnormal mitochondria suggesting muscle damage and/or remodelling (Fig. 7A and B). This could cause oxidative stress followed by activation (phosphorylation) of p38 MAPK and autophagy (21). Indeed there is an increase in p38 MAPK and increased myosin heavy chain expression (Fig. 7C and Supplementary Fig. 6).

Patients with WMS are reported to have thickened skin. Although we did not fully characterize the skin we observed that ADAMTS10 predominantly localized to the epidermis and hair follicles in wild-type mouse skin and ADAMTS10^S236X/S236X^ mice have increased skin thickness and convolution at the dermal–epidermal junction (Supplementary Fig. 7). Embryonic fibroblasts were derived from the skin of E13.5 wild-type and ADAMTS10^S236X/S236X^ mice to analyse changes in expression and signalling pathways. In ADAMTS10^S236X/S236X^ mouse embryonic fibroblasts (MEFs) there was decreased pSMAD 1/5/8 indicative of reduced BMP signalling and reduced expression of BMPs 2,4,5,7 but no significant change to TGFβ signalling (Fig. 8A and B). Furthermore, there was decreased expression of syndecan-4 in both ADAMTS10^S236X/S236X^ MEFs and skin (Fig. 8C) and as a focal adhesion receptor might lead to a disruption of focal adhesion function and/or formation, cell–cell interactions and cell signalling. These data are consistent with the reported role for ADAMTS10 in focal adhesion formation where it has been shown to interact with syndecan-4 and ADAMTS10 depletion disrupts focal adhesion formation (13). Immunofluorescence data from MEFs suggests that the truncated ADAMTS10 protein is secreted. Some protein is incorporated into fibrillar structures but there are also signs of amorphous protein in and outside of the cell (Fig. 8D).

## Discussion

WMS patients have short stature, ocular abnormalities, thick skin and are hypermuscular. Many of these phenotypes are present in the ADAMTS10^S236X/S236X^ mice indicating this mouse to be a good model for WMS. The homozygous knock-in mice are smaller (both lighter and shorter) from birth and remain so to adulthood, due at least in part to shorter long bones. The mice have abnormalities to the ciliary apparatus and increased microfibrils near the CB which are Fbn2 rich and the skeletal muscle also has more Fbn2 deposition. The epidermis of the skin is thicker with increased ruffling at the epidermis. The mice have increased muscle mass when normalized for their smaller size and there are increased numbers of smaller muscle fibres compared to wild type, consistent with hypermuscularity.

ADAMTS10 has been shown to interact with Fbn1 and is thought to facilitate fibrillin microfibril deposition as demonstrated by exogenous addition of ADAMTS10 to fetal nuchal ligament cells (2). Furthermore, WMS patients and Fbn1 WMS mice have abundant microfibrils in skin (8) and the CZ in the ADAMTS10^S236X/S236X^ mice has increased numbers of fibrillin microfibrils. However, it is not clear whether ADAMTS10 has a direct role in the formation of microfibrils and microfibril bundles or influences expression of fibrillins. Individual microfibrils purified from skin have normal morphology and the zonule fibres and bundles of microfibrils in the CZ also have apparently normal structures suggesting at least in these tissues that ADAMTS10 is not required for their normal assembly. More likely expression of fibrillin could be increased by altered cell–cell signalling perhaps due to disrupted cell junctions/cell adhesion indicated by the changes observed in syndecan-4 expression and disrupted ZO-1-rich tight junctions.

Fbn2 is the dominant isoform in early eye development (22) and in the adult little Fbn2 is present in the CZs (Fig. 5), however the adult ADAMTS10^S236X/S236X^ mice have abundant Fbn2 staining in the CZ. Moreover, in the skeletal muscle of the WT mice no Fbn2 staining is observed but the ADAMTS10^S236X/S236X^ mice have abundant Fbn2 staining in common with the CZs. The Fbn2^−/−^ mouse has plentiful but disorganized microfibrils suggesting that Fbn1 and Fbn2 can interchange to some extent (22,23). Indeed ADAMTSL2 can regulate the fibrillin isoform composition of microfibrils (24). These findings suggest that ADAMTS10 is involved in the developmental expression switch from Fbn2 to Fbn1 in the eye and muscle. One explanation is that Fbn2 expression continues into adulthood perhaps due to altered cell–cell signalling in the ADAMTS10^S236X/S236X^ mice, or perturbation to the tissue morphology alters the balance of cells expressing the relative fibrillin isoforms.

Indeed Fbn2 expression would normally decrease postnatally with a concomitant increase in Fbn1 expression, with the valleys between ciliary processes in the non-pigmented ciliary epithelium having the highest Fbn1 expression (22). However, the ADAMTS10^S236X/S236X^ mice have altered development of the ciliary apparatus with reduced CB area and fewer ciliary folds which could perturb the balance of Fbn1/Fbn 2 expression. The presence of both Fbn1 and Fbn2 isoforms results in the increased numbers of microfibrils in the CZs in particular in the region close to the CB which is the main site of expression. WMS patients also have abnormalities in their CB and CZ leading to ectopia lentis (20), the only clinical manifestation in common with MFS. These abnormalities could be due to altered proliferation of non-pigmented ciliary epithelium cells and/or early cell cycle withdrawal and disrupted commitment to terminal differentiation possibly in a BMP-dependent manner (22,25).

ADAMTS10 mice have shorter long bones and the changes in width of growth plate zones suggest subtle changes in growth plate dynamics. Altered chondrocyte differentiation, as shown by increased proliferation and slightly expanded HZ, could lead to an increased load for hypertrophy and in turn slower chondrocyte apoptosis and mineralization. In MEFs there is reduced BMP expression and signalling, coupled with altered proliferation in the growth plate which could contribute to this phenotype. Indeed, mice lacking BMP-6, which is expressed by osteoblasts and chondrocytes in the growth plate, have a reduction in the size of the long bones and BMP-6 has a role in growth plate function (26). Moreover BMP-5 deficient mice have shorter long bones with changes in the deceleration of chondrocyte proliferation and initiation of hypertrophic differentiation suggesting that BMP-5 inhibits the differentiation of chondrocytes into hypertrophic cells (27,28). The pro-domain of BMP-5 binds to Fbn1 and Fbn2 which may regulate the extracellular activity of the growth factor as has been demonstrated for proBMP7 (29,30), suggesting that changes in gene expression or extracellular interactions could perturb the balance of BMP signalling.

WMS patients are observed to be of a muscular build or hypermuscular (31,32) but it is not known if their increased muscle mass results in increased strength or muscle function. We found that in skeletal muscle ADAMTS10 was localized to the muscle niche and its disruption perturbed ZO-1-rich tight junctions. The ADAMTS10^S236X/S236X^ mice have increased skeletal muscle mass and myofibres but myofibres are smaller, which is of particular interest, as the number of fibres per given muscle in a given species is essentially determined at birth (33). Further growth and hypertrophy depend upon fusion of satellite cells to the fibre and increased synthesis of actomyosin. This is a rare situation where the number of fibres is almost doubled at 4 weeks and most likely at birth. Thus, this model may offer a useful tool to investigate how the number of fibres is determined, as altered remodelling of the extracellular matrix may lead to myofibre duplication during early myogenesis.

It is unclear what contributes towards the muscle growth and engagement of satellite cells but there are signs of cell stress in the myofibres and changes in gene expression include BMP/GDF growth factors. Activation of p38 signalling to instigate myogenesis can be triggered by a number of stimuli including growth factors and cell–cell contacts (34) and myogenic differentiation can result in increased muscle mitochondria volume (35). The importance of BMP signalling in skeletal muscle differentiation has been shown previously in the Fbn2 null muscle where increased numbers of muscle cells with centrally located nuclei were due to a delay in differentiation caused by excess activation of BMP signalling (36). These findings and the persistence of Fbn2 in the adult ADAMTS10^S236X/S236X^ mice are consistent with the opposing clinical features of the Marfanoid and the short-stature syndromes linked to fibrillin mutation.

In summary, the ADAMTS10^S236X/S236X^ mouse is a good model for WMS presenting many similar phenotypes to patients. Truncation of ADAMTS10 does not affect the formation of fibrillin microfibrils or microfibril bundles but does result in gene expression changes which could be as a consequence of altered cell–cell signalling perhaps due to disrupted cell junctions/cell adhesion.

## Materials and Methods

### Animal experiments

All animal procedures were performed under license in accordance with the UK Home Office Animals Scientific Procedures Act 1986 and the Association for Research in Vision and Ophthalmology (ARVO) Statement for the Use of Animals in Ophthalmic and Vision Research and were approved by the University of Manchester Animal Welfare and Ethical Review Body. Mice were asphyxiated with CO_2_ and tissues were harvested for analysis.

### Generation of S236X mutant mice

To generate the S236X transgenic mouse we identified Single guide RNA (sgRNAs) targeting this region (*AGTCAGCAGAGAGCGCTATG-TGG and GGCACTTACAATGTTCATGA-TGG*) were subcloned into the pUC57-sgRNA vector (https://www.addgene.org/51132/) using BsaI overhangs according to protocols in (37). Sequence verified vector was linearized by SalI digestion and sgRNA was synthesized *in vitro* (HiScribe, NEB, as per manufacturer’s instructions), before purification (Megaclear, Ambion), elution in embryo microinjection buffer (10 mM Tris (pH 7.5), 0.1 mM ethylenediaminetetraacetic acid (EDTA) (pH 8.0) and 100 mM NaCl) and quantification by nanodrop. A single stranded DNA (ssDNA) repair template was designed, with 50 bp 5′ and 3′ flanking homology, to excise 106 bp of this exon, allowing a size difference for simpler genotyping of mouse colonies and simultaneously introduce a premature stop codon, was ordered with PAGE purification (CCCCTGGGGAATGAATCTGAGCGAGGCCAGCTGGGCCTGAAGAGATCAGTGTAAGTGCCTTCCCCTTCCTAGGCCTGGAGTCCTGAGGACCTGTAGCTAG, Integrated DNA Technologies) and resuspended in embryo microinjection buffer. An injection mix of the two sgRNA (40 ng/μl each), Cas9 mRNA (100 ng/μl) and the ssDNA repair template (100 ng/μl) was prepared and directly microinjected into B6D2F1 (Envigo) zygote pronuclei using standard protocols. Zygotes were cultured overnight and the resulting 2 cell embryos surgically implanted into the oviduct of day 0.5 post-coitum pseudopregnant mice.

Potential founder mice were screened by PCR after extraction of genomic DNA from ear punches (Red-N-Extract kit, Sigma) using CK01 Geno F1 CTGAAGAGCATGGACACTGTC and CK01 Geno R1 TTATTGTTCTGGCCCCTGGT, pups demonstrating predicted size differences were taken forward for sequencing by pCR-Blunt cloning followed by Sanger sequencing. Two founder mice were identified and then back-crossed to C57BL/6 J wild-type mice to assess germline penetrance. After two crosses and once germline penetrance was confirmed, heterozygous mice were outcrossed generating both WT and HOM littermates, which were directly compared in each experiment.

### Antibodies

Primary antibodies used for immunofluorescence microscopy and western blotting were ADAMTS10 (custom synthesized pro-domain specific rabbit, Biomatik, 1:50), rat anti-ZO-1 (Millipore; T11, 1:200), rabbit anti-Laminin (Abcam, ab11575, 1:100), rabbit anti-PECAM (Abcam, ab 28364, 1:100), rat anti-BrdU (Sigma-Aldrich, RPN201,1:200), goat anti-MAGP1 (Santa Cruiz, sc-166075, 1:200), rabbit anti-Pp38 antibody (Cell Signalling 9211, 1:500) and rabbit anti-p38 antibody (Cell Signalling 8690, 1:500), rabbit anti-pSMAD1/5/8 (Millipore, AB3848-I, 1:500), rabbit anti-SMAD1 (Cell Signalling 9743, 1:500), rabbit anti-Fbn1 and rabbit anti-Fbn2 (1:500). Polyclonal rabbit anti-Fbn1 and anti-Fbn2 antisera were raised against the C-terminally double-strep-tagged N-terminal recombinant human Fbn2 polypeptide rF86 (Gln^29^-Asp^535^) and C-terminally His_6_-tagged rF90 representing the N-terminal half of Fbn1 expressed in 293EBNA cells (38,39). Antisera were purified before usage by affinity chromatography on a column with antigen coupled to cyanogen bromide-activated Sepharose (GE Healthcare). Secondary antibodies used were purchased from ThermoFisher Scientific: Donkey anti-rabbit Alexa Fluor 488 (A21207, 1:400), Donkey anti-rat Alexa Fluor 594 (A21209, 1:400), Donkey anti-goat Alexa Fluor 594 (A11058, 1:400) or Li-COR: Donkey anti-rabbit (IRDye 680RD 925-68073, 1:10000), Donkey anti-rabbit (IRDye 800RD 925-32213, 1:10000). Tissues were stained using the blue or grey 4′,6,-Diamidine-2′-Phenylindole dihydrochloride (DAPI), red or grey (594 nm) green or grey (488) channels, then false coloured.

### Skeletal measurements

Culled mice were placed on X-Ray hyperfilm (GE healthcare, GZ28906850) and X-Rayed using a Flaxitron X-ray specimen radiography system (Flaxitron MX-20). Measurements of the whole body (excluding tail), femur and tibia length were taken manually from the scanned X-Ray films.

### Tissue processing

Hind limb, eye and skin tissue samples were dissected and fixed overnight in ice-cold 4% paraformaldehyde (PFA) (w/v) in 1 x Phosphate buffered saline (PBS) and then stored in 70% ethanol until processing. Hind limbs were fixed in either 4% PFA or 95% ethanol/5% acetic acid (v/v) and decalcified in 0.8 M EDTA pH 7.4 for a period of at least 1 week. PFA and ethanol/acetic acid fixed tissue samples were embedded in paraffin wax and sectioned sagittally using a cool-cut HM 355 S microtime (MicRom) generating 5 μm-thick sections. Muscle samples were cryopreserved in liquid nitrogen and isopentane and stored at −80°C until processing. For Immunofluorescence (IF) labeling, 10 uM cross sections were generated using Microm HM560 automated cryostat (Thermo Scientific). For electron microscopy, muscle and eye samples were fixed in 4% PFA, 2.5% Glutaraldehyde, 0.1% 4-(2-hydroxyethyl)-1-piperazineethanesulphonic acid (HEPES). TA, G and Q were dissected and weighed. Muscle samples were cryopreserved and subjected to IF staining for Laminins, ADAMTS10, ZO-1, PECAM and DAPI. Measurements from around 1 000 TA myofibres from 5 areas were taken using ImageJ, nuclei were counted using the particle analysis-nuclei count plugin.

### Growth plate analysis

H&E and BrdU staining was performed for growth plates as described previously (40,41). Growth plate zone widths were measured on images of known magnification as described previously (40). The RZ was measured from the top to the beginning of the PZ. The beginning of the proliferative zone was defined as the point at which the individual round chondrocytes flattened out and arranged into columns. The start of the HZ was defined as the point at which proliferative chondrocytes rounded up and enlarged. The end of the HZ was defined as the vascular invasion front. For each animal the data from at least five separate sections, spaced a minimum of 50 μm apart were averaged.

### MEFs

MEFs were isolated from 3 WT and 3 HOM E13.5 embryos according to a standard protocol (42). MEFs were cultured in Dulbecco’s Modified Eagle’s Medium (DMEM; Sigma-Aldrich and supplemented with 10% (v/v) fetal calf serum (FCS; Thermo Fisher Scientific), 1% L-glutamine, 100 U/ml penicillin/streptomycin at 37°C in 5% CO_2_ for 4–5 days in 6 well plates. MEF samples were subjected to Reverse transcription-PCR (RT-PCR) to detect syndecan-4 and Bmp2, 4, 5, 7 mRNA expression levels and Western Blotting to detect phosphorylation of SMAD 1/5/8 and SMAD2/3.

### Ribonucleic acid (RNA) isolation and real-time PCR analysis

Mouse tissues were homogenized in 500 ul of RNA lysing buffer in homogenizer (MP Biomedicals, FastPrep 24 5G) using metal beads (MP Biomedicals, 116925100) 2 x 60s cycle and then centrifuged at 13 000 rpm for 10 min. at 4°C. Supernatant was taken to a fresh tube and RNA was isolated using ReliaPrep™ RNA Cell Miniprep System (Promega). 250 ng to 1 ug RNA was subjected to first strand cDNA synthesis using Bioline kit. RT-qPCR analysis was carried out using CFX96/384 instruments (Bio-Rad) and the GoTaq qPCR Mastermix Kit (Promega). RT-qPCR primer sequences are listed in Supplementary Table 1. Expression analysis used CFX Manager Software v3.1 (Bio-Rad), with samples normalized to a combination of TATA box-binding protein and glyceraldehyde 3-phosphate dehydrogenase expression.

### Serial blockface SEM

WT and homozygous eyes were prepared for serial block face scanning electron microscopy (SBF-SEM) as previously described (43,44). Eyes were oriented so the CZ to lens capsule was imaged in longitudinal cross section. Regions of interest were selected and scans taken using a Quanta Field emission gun (FEG) 250 (FEI) equipped with a Gatan 3View ultramicrotome at 3.8 kV collecting back scattered electrons. Data sets of 400 slices were collected and sections of ∼100 nm in thickness were removed from the sample blocks after each scan.

### Immunofluorescent labelling

Paraffin section staining was performed using a standard protocol including deparaffinization with: Xylen 1 for 3 min and Xylen 2 for 3 min and rehydration with ethanol 100% for 2 min, ethanol 90% for 2 min, ethanol 70% for 2 min and dH_2_O for 2 min followed by antigen retrieval with 1 mg/ml Trypsin. For cryosections no antigen retrieval step was required. BrdU assay was performed according to (41), however anti-rat Alexa Fluor 488 was used instead of HRP-conjugated secondary antibody. Images were collected at room temperature on an Olympus BX51 upright microscope using 20x or 60x objectives and captured using a Coolsnap ES camera (Photometrics) through MetaVue Software (Molecular Devices). Specific band pass filter sets for DAPI, FITC, Texas Red were used to prevent bleed through from one channel to the next. Images were processed and analysed using ImageJ (http://rsb.info.nih.gov/ij).

## Statistical analysis

The results were analysed by two-tailed unpaired Student t-test for statistical significance using GraphPad Prism 6.0 software.

## Supplementary Material

Supplementary Data
