# Supplementary material for: ADAMTS10-mediated tissue disruption in Weill–Marchesani syndrome
Source: Hum Mol Genet. 2018 Jul 27;27(21):3675–87. doi: 10.1093/hmg/ddy276 (PMC6196651; doi:10.1093/hmg/ddy276)
Supplement: Supplementary Data [file ddy276_supp.pdf]

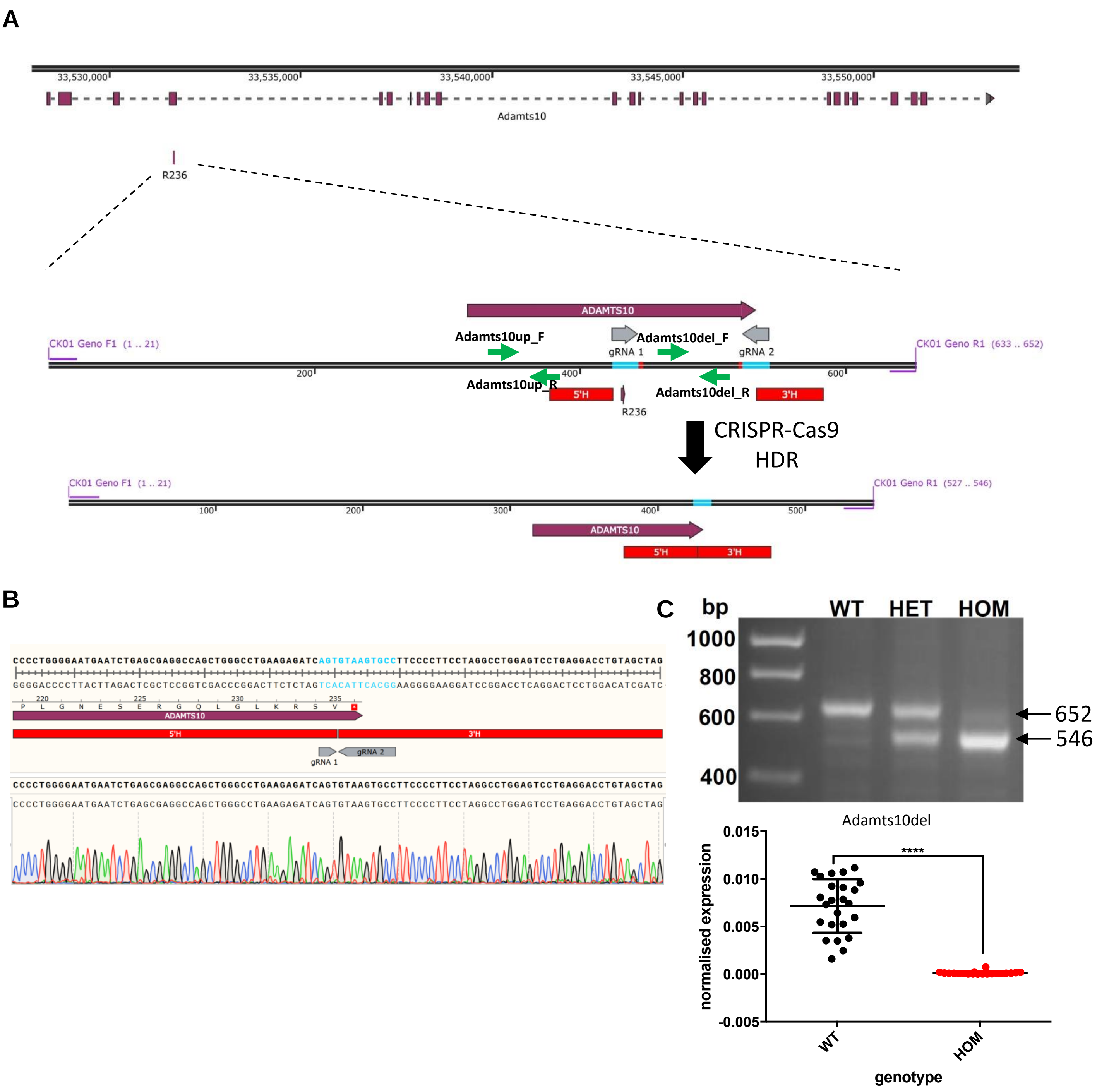

Supplementary Fig. 1 Generation of the WMS ADAMTS10$^{S236X/S236X}$ mutant mouse using CRISPR technology. A) Schematic of target region, with sgRNA binding sites indicated, and homology arms for 106bp sequence excision. Green arrows show the positions of the forward (F) and reverse (R) qPCR primers with Adamts10up upstream of the mutation and Adamts10del within the 106 bp deletion. **B)** Sequencing of founder with loss of sequences and integration of premature stop codon. **C)** PCR Genotyping of WT (652 bp band), HET (652 bp and 546 bp band) and HOM (546 bp band) mice. The RT-PCR graph using primers targeting the 106 bp deletion confirm this region is deleted.

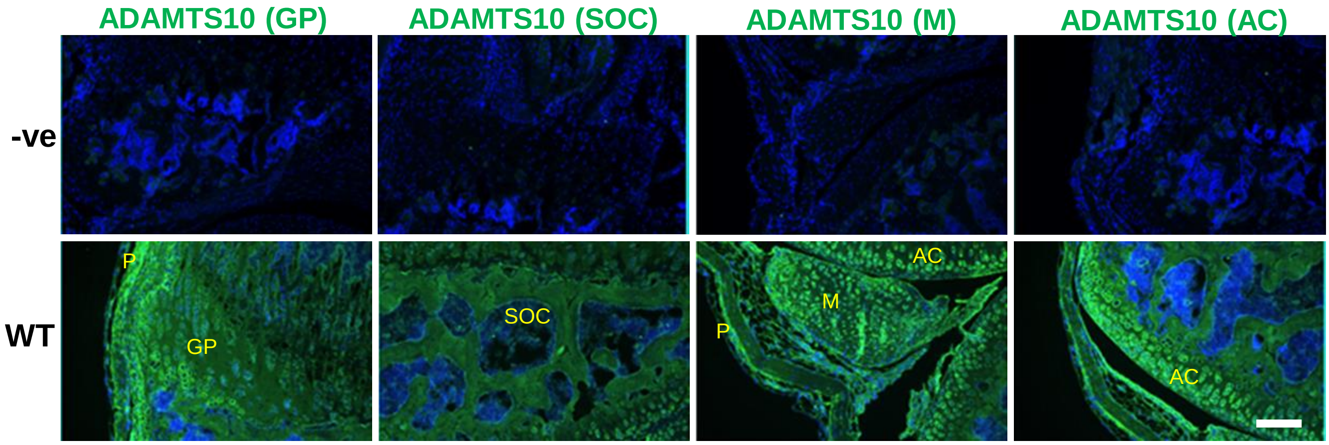

Supplementary Fig. 2 Localisation of ADAMTS10 in the mouse knee joint. IF microscopy of the 3 week-old mouse knee joint structures stained with ADAMTS10 (green) and DAPI (blue) in the growth plate – GP, perichondrium - P, meniscus – M, secondary ossification centres – SOC, articular cartilage – AC. ADAMTS10 is predominantly expressed in the articular cartilage, perichondrium and meniscus but also present in the secondary ossification centres and growth plate. No background staining was present on negative controls (-ve), scale bar=200 µm.

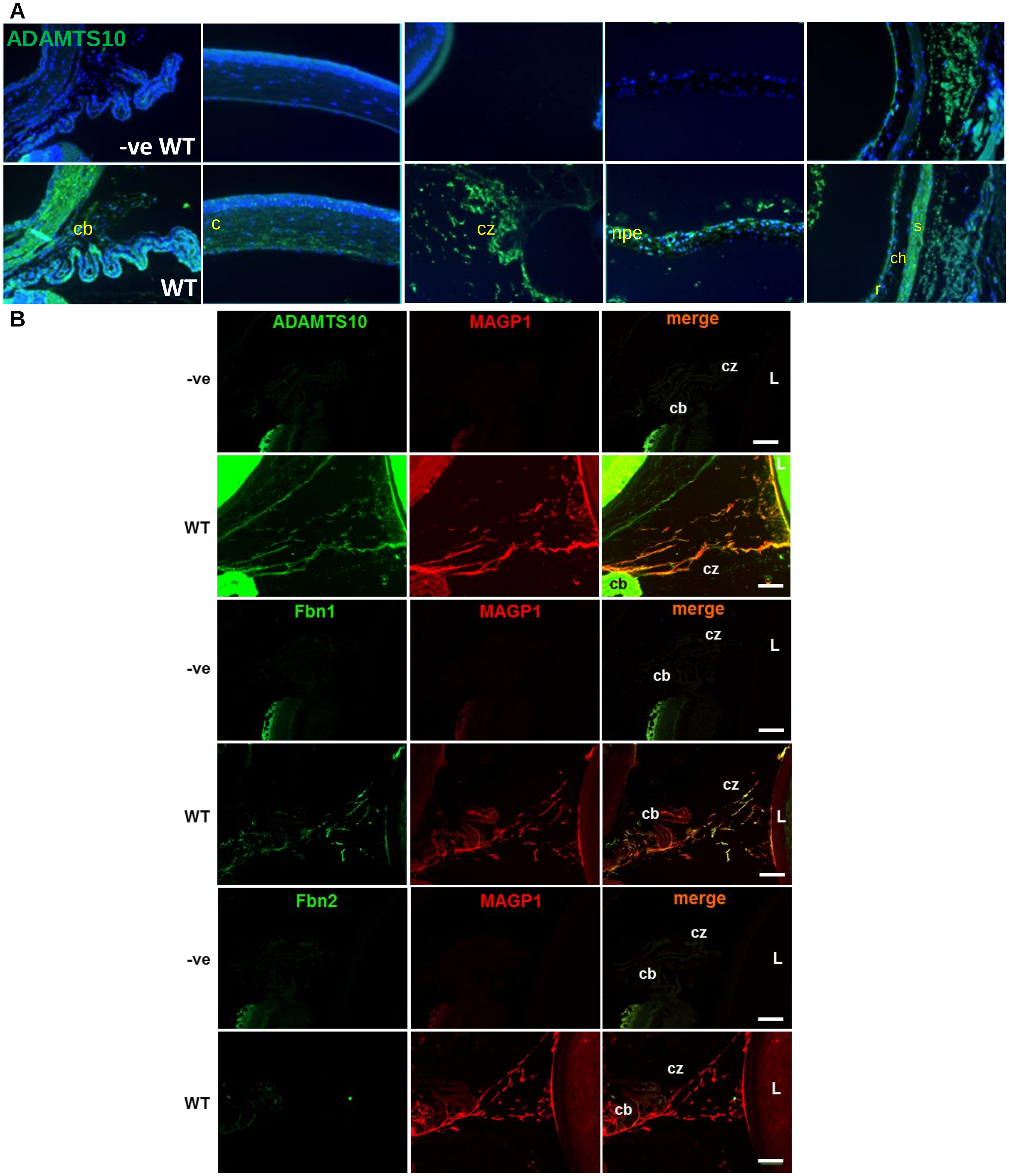

Supplementary Fig. 3A) Localisation of ADAMTS10 protein within the mouse eye structures. Immunohistochemical staining of ADAMTS10 protein (green) and DAPI (blue) in the 3 month-old WT ciliary body (cb), ciliary zonule (cz), non-pigmented epithelium (npe), retina (r), sclera (s), choroid (ch). No ADAMTS10 (green) staining detected in the cb and cz with some autofluorescent background staining around the sclera in the negative control (-ve), scale bar=50 µm. **B)** Negative control (–ve) images for IF images shown in Fig. 5.

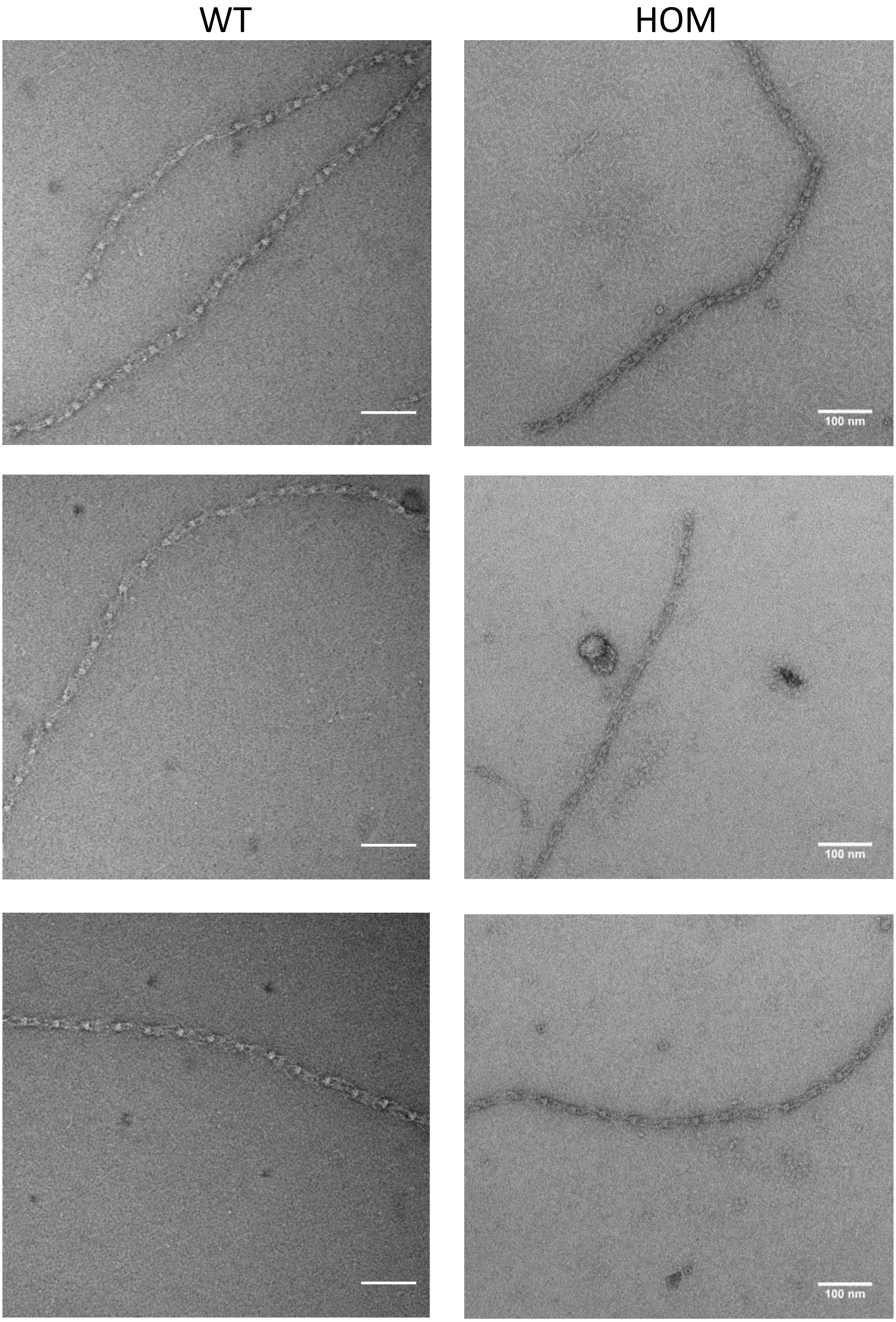

Supplementary Fig. 4. Negatively stained TEM images of microfibrils purified from WT and HOM skin. Scale bars = 100 nm.

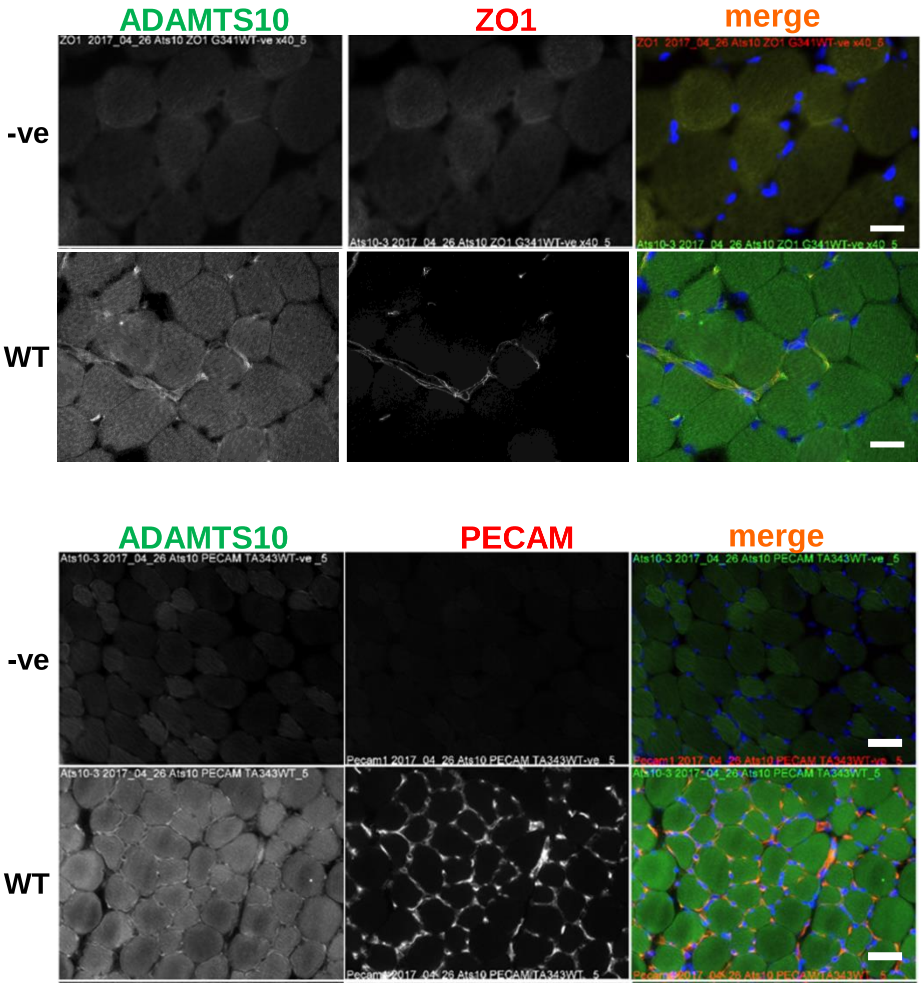

Supplementary Fig. 5 Negative control (-ve) images for IF images shown in Fig. 6D and E. Scale bars = 50 μm.

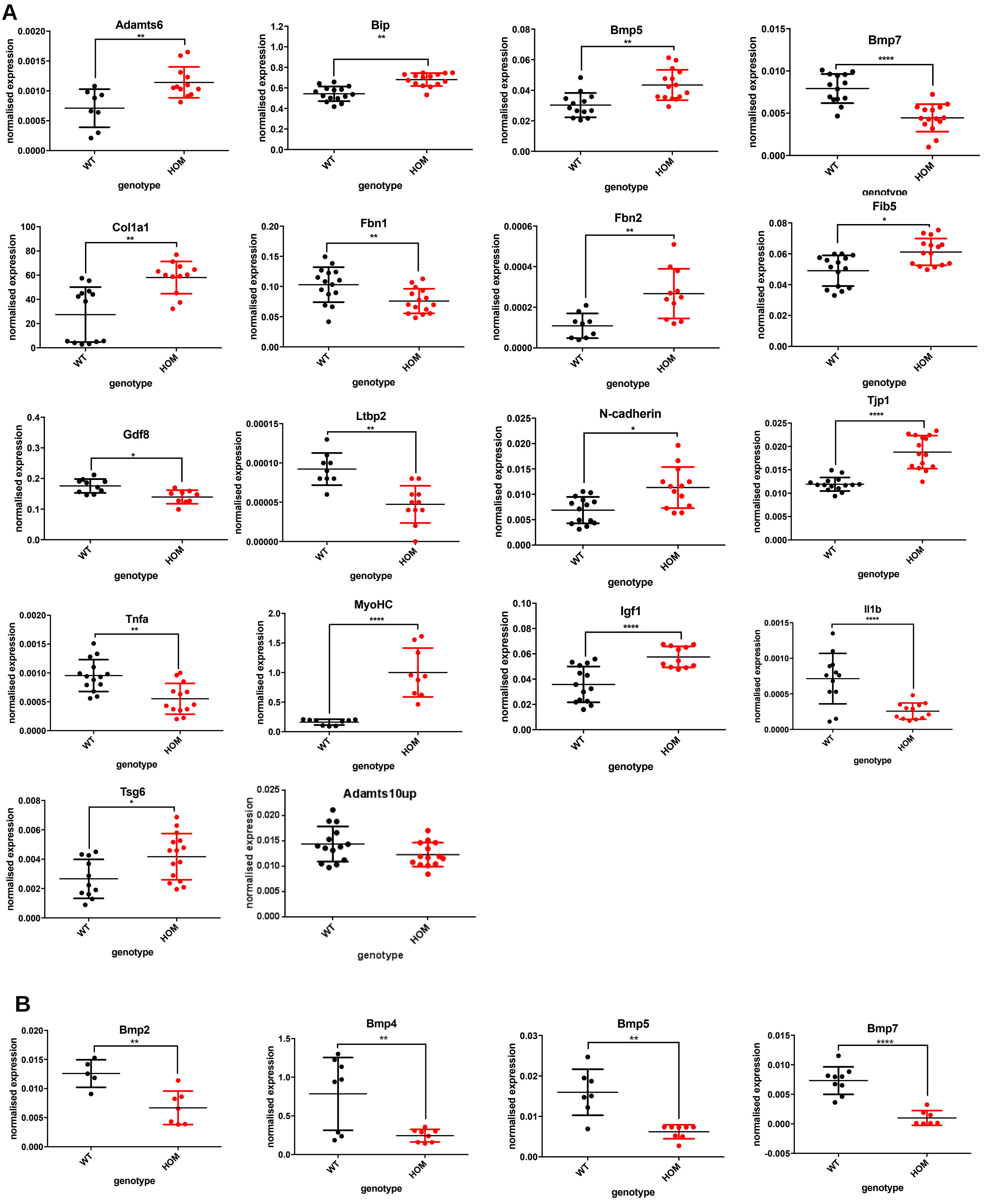

Supplementary Fig. 6 RT-PCR graphs showing altered gene expression. A) 4 week-old muscles, **B)** 2D MEFs day 4. As shown in Supplementary Figure 1A, Adamts10up primers are upstream of the mutation as shown in Supplementary Figure 1A.

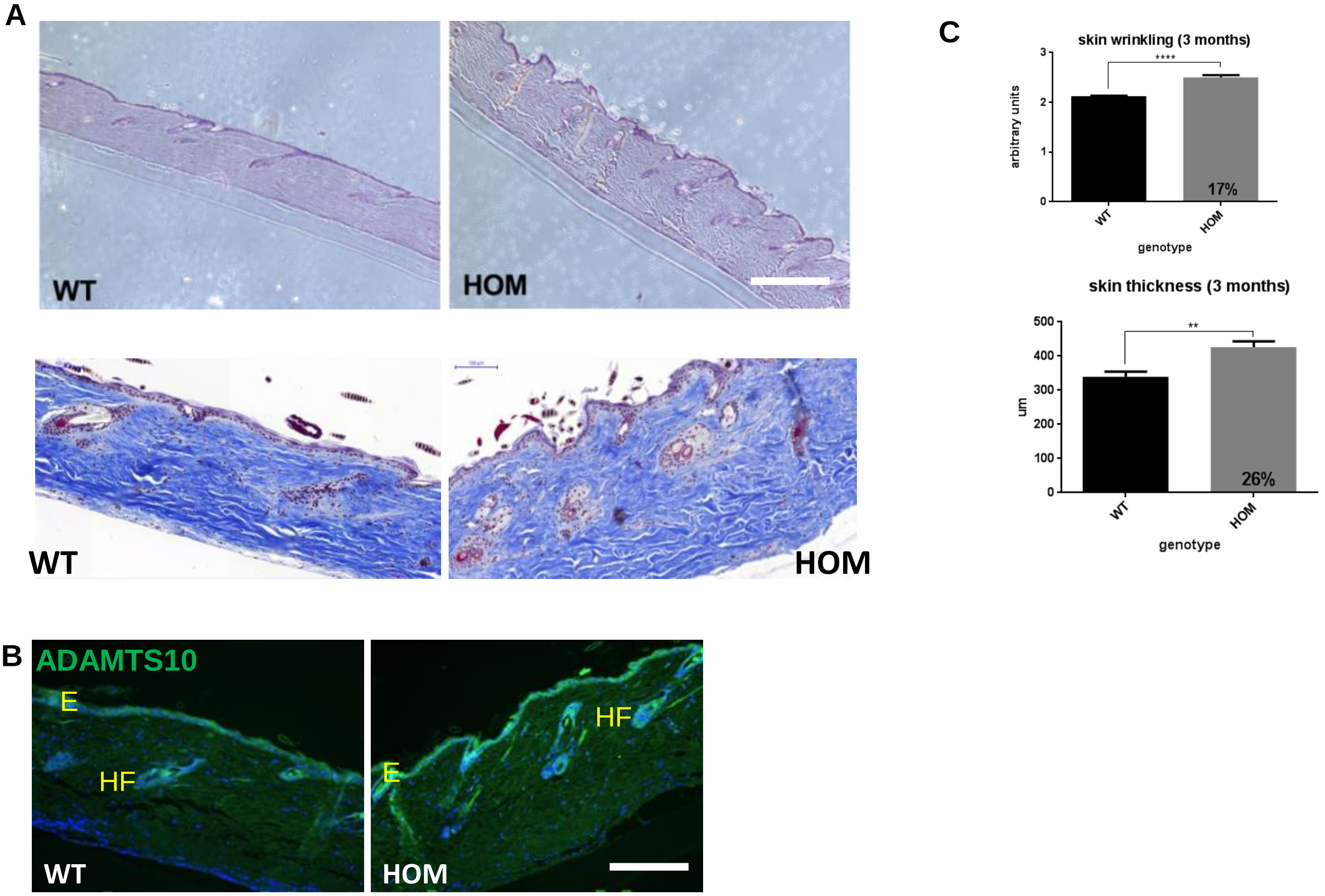

Supplementary Fig. 7. Increased skin thickness in WMS ADAMTS10$^{S236X/S236X}$skin. A) H&E (top panel) and Trichrome Masson's (bottom panel) staining of the WT and HOM skin sections. Scale bar =500 µm (top) and 200 µm (bottom). **B)** IF staining of ADAMTS10 (green) and DAPI (blue) of the WT and HOM skin sections with predominant localisation in the epidermis (E) and hair follicles (HF), scale bar=200 µm **C)** Increased skin ruffling and skin thickness in the HOM animal. Statistical significance was calculated using two-tail unpaired Student's test in GraphPad Prism V6. Asterisk indicate P values where **P ≤ 0.01; ****P ≤ 0.0001.

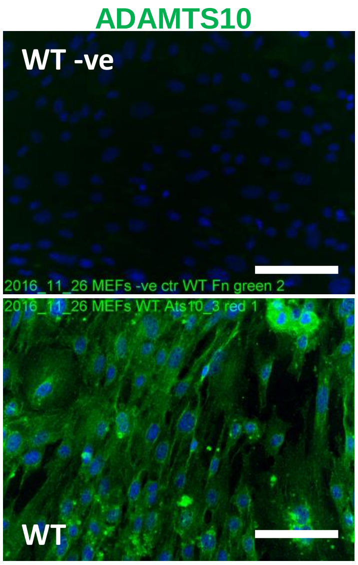

Supplementary Fig. 8 Localisation of ADAMTS10 protein within MEFs negative control images (-ve) for IF images shown in Fig. 8D. Scale bar=100 µm.

Supplementary Table 1. RT-PCR primer sequences:

gene	primer sequence
Adamts10up F	gagagtggcccccatgtagt
Adamts10up R	tcatctctcactccacaggct
Adamts10del F	ccaaccattgggggcaagta
Adamts10del R	agccaggtggacagtcattg
Adamts6 F	aggagctcgctctgtgtaga
Adamts6 R	ccctatactggaggtggggt
Bip F	ggcaccttcgatgtgtctcttc
Bip R	tccatgacccgctgatcaa
Bmp5 F	ttcaaggcaagcgaggtact
Bmp5 R	tgcaggcttgtttttgttca
Bmp7 F	ggaagcatgtaagggttcca
Bmp7 R	ttcctggcagacatttttcc
Col1a1 F	cacctacagcacccttgtgg
Col1a1 R	gggaggtcttggtggttttg
Fbn1 F	ttgcgagtgtgagattggct
Fbn1 R	tggtggcatccagacactca
Fbn2 F	atacaatgtcggcaaagcct
Fbn2 R	atggtcttaaagtcagctgtccc
Fib5 F	ccgataccctggtgcctatt
Fib5 R	gcactgataggccctgtttg
Fn F	tgataccgttgtcccagaggt
Fn R	aggccgatgcttgaatcagt
Gdf8 F	tgacagcagtgatggctctt
Gdf8 R	aagtcagactctgtaggcatgg
Igf1 F	gaagcctacaaaagcagccc
Igf1 R	tagggacggggacttctgag
Il1b F	ccttgtgcaagtgtctgaagc
Il1b R	catcactgtcaaaaggtggca
Ltbp2 F	gagcctcccaaatggatacaga
Ltbp2 R	ctcatcgatatcagtacagtagtct
MyoHC F	ccaagggcctgaatgaggag
MyoHC R	gcaaaggctccaggtctgag
N-cad F	cagccccttctcaatgtgaaat
N-cad R	cttgaaatctgctggctcgc
Sdc4 F	ccggagagtcgattcgagag
Sdc4 R	gggagggctccagagaagta
Tjp1 F	aagcgcagcccacaagctatt
Tjp1 R	tgaggcttctgctttctgttg
Tnfa F	ttctatggcccagaccctca
Tnfa R	tggtttgctacgacgtggg
Tbp F	gcacaggagccaagagtgaa
Tbp R	tagctgggaagcccaacttc
Gapdh F	ggctcatgaccacagtccat
Gapdh R	atcacgccacagctttccag
